# Antibodies Reactive to Commensal *Streptococcus mitis* Show Cross-Reactivity With Virulent *Streptococcus pneumoniae* Serotypes

**DOI:** 10.3389/fimmu.2018.00747

**Published:** 2018-04-16

**Authors:** Sudhanshu Shekhar, Rabia Khan, Daniela M. Ferreira, Elena Mitsi, Esther German, Gro Herredsvela Rørvik, Dag Berild, Karl Schenck, Keehwan Kwon, Fernanda Petersen

**Affiliations:** ^1^Department of Oral Biology, Faculty of Dentistry, University of Oslo, Oslo, Norway; ^2^Department of Clinical Sciences, Liverpool School of Tropical Medicine, Liverpool, United Kingdom; ^3^Faculty of Medicine, Institute of Clinical Medicine, University of Oslo, Oslo, Norway; ^4^Infectious Diseases Group, J. Craig Venter Institute, Rockville, MD, United States

**Keywords:** *Streptococcus pneumoniae*, *Streptococcus mitis*, antibody, antigens, cross-reactivity

## Abstract

Current vaccines against *Streptococcus pneumoniae*, a bacterial species that afflicts people by causing a wide spectrum of diseases, do not protect against all pneumococcal serotypes. Thus, alternative vaccines to fight pneumococcal infections that target common proteins are under investigation. One promising strategy is to take advantage of immune cross-reactivity between commensal and pathogenic microbes for cross-protection. In this study, we examined the antibody-mediated cross-reactivity between *S. pneumoniae* and *Streptococcus mitis*, a commensal species closely related to *S. pneumoniae*. Western blot analysis showed that rabbit antisera raised against *S. mitis* reacted with multiple proteins of virulent *S. pneumoniae* strains (6B, TIGR4, and D39). Rabbit anti-*S. pneumoniae* IgG antibodies also showed binding to *S. mitis* antigens. Incubation of rabbit antisera raised against *S. mitis* with heterologous or homologous bacterial lysates resulted in marked inhibition of the developments of bands in the Western blots. Furthermore, plasma IgG antibodies from adult human volunteers intranasally inoculated with *S. pneumoniae* 6B revealed enhanced *S. mitis*-specific IgG titers compared with the pre-inoculation samples. Using an on-chip protein microarray representing a number of selected membrane and extracellular *S. pneumoniae* proteins, we identified choline-binding protein D (CbpD), cell division protein (FtsH), and manganese ABC transporter or manganese-binding adhesion lipoprotein (PsaA) as common targets of the rabbit IgG antibodies raised against *S. mitis* or *S. pneumoniae*. Cumulatively, these findings provide evidence on the antibody-mediated cross-reactivity of proteins from *S. mitis* and *S. pneumoniae*, which may have implications for development of effective and wide-range pneumococcal vaccines.

## Introduction

*Streptococcus pneumoniae* is a Gram-positive bacterial species that resides in the nasopharynx and oral cavity of humans and causes a wide spectrum of diseases, including pneumonia, meningitis, and septicemia (1). On the other hand, *Streptococcus mitis*, a commensal that exhibits genetic and ecological similarity with *S. pneumoniae*, is one of the most abundant members of the oral microbiota and rarely causes disease (2). Clinic-epidemiological studies indicate that *S. pneumoniae* is the fourth most frequent cause of lethal infections and inflicts death on more than a million children under the age of 5 years worldwide (3, 4). In addition, immunocompromised and elderly individuals are highly susceptible to *S. pneumoniae* infections (3, 5). Although current pneumococcal vaccines, both conjugated (PCV7, 10, and 13) and unconjugated (PPSV23), have reduced the occurrence of diseases, they are unable to confer protective immunity against all *S. pneumoniae* serotypes (more than 90) (6, 7). Recent reports showing replacement by non-vaccine serotypes pose grave concern (7). Therefore, novel prophylactic strategies are needed to develop better vaccines that could provide long-term protection against all pneumococcal serotypes.

Understanding host immunity to pneumococcal infections is crucial for identifying immune targets for vaccine development. Several studies involving mouse models of *S. pneumoniae* indicate that both arms of the adaptive immunity (antibody and T cell) play an important role in mounting protective immunity to infection (8–14). In a mouse model of nasopharyngeal colonization by *S. pneumoniae*, it was shown that mice deficient in CD4+ T cells or IL-17 had a reduced ability to clear streptococcal carriage compared with control mice (12). By contrast, protective immunity to septicemia after previous colonization is conferred by antibody-mediated (IgG and IgA) immunity (14). This suggests that the type of protective immune response against pneumococcal infections is largely influenced by the site of infection. Using a murine model of nasopharyngeal colonization by *S. pneumoniae* EF3030 followed by lung infection, Wilson et al. recently showed that colonization-induced protection in mice deficient in B cells, CD4+ T cells or IL-17 is impaired, suggesting that naturally acquired immunity to *S. pneumoniae* lung infection requires both humoral and cellular immunity (13). This has also been confirmed in controlled human infection studies with pneumococcus. Following experimental colonization, volunteers protected against subsequent re-colonization with the same strain presented increased pneumococci-specific antibodies as well as CD4+ T cells (8). On the other hand, how the host responds to *S. mitis* is poorly understood. Salivary IgA antibodies reactive to *S. mitis* antigens have been reported in human infants (15–17). Our recent study has shown that human CD4+ T cells, which express IL-17 (Th17 cells) and are reactive with *S. mitis*, also respond to pneumococcal proteins when cocultured with antigen-presenting cells pulsed with *S. pneumoniae* (18).

Currently available pneumococcal vaccines provide immunity that is mediated by serotype-specific antibody responses, which has limitations as this protection is restricted to only 7–23 serotypes. In addition, capsular polysaccharide vaccines are more protective against invasive pneumococcal diseases than non-invasive diseases, such as otitis media (5, 6). A novel approach to deal with these limitations and provide broader serotype coverage could be to exploit the immune cross-reactivity between commensal and pathogenic microbes. The antibodies generated against commensal microbes have been reported to cross-react with pathogens (18–20). Although a few studies have examined the serological cross-reactivity of polysaccharide antigens between *S. mitis* and *S. pneumoniae*, it remains unclear whether antibodies specific to *S. mitis* cross-react with pneumococcal proteins (21–23). In this study, we hypothesized that antibodies elicited against *S. mitis* and *S. pneumoniae* show cross-reactivity to *S. pneumoniae* and *S. mitis* proteins, respectively. To test this hypothesis, we examined the ability of antibodies raised against *S. mitis* or *S. pneumoniae* to cross-react with *S. pneumoniae* and *S. mitis* and attempted to identify pneumococcal protein antigens that react with the antibodies. Our findings shed light on the antibody-mediated cross-reactivity between *S. mitis* and *S. pneumoniae*. This may have implications for designing effective prophylactic strategies against pneumococcal infections. *S. mitis*-based vaccines can induce the production of antibodies reactive to *S. pneumoniae* proteins, resulting in serotype-independent protection against pneumococci.

## Materials and Methods

### Bacterial Strains, Culture, and Lysis

*Streptococcus mitis* strain used was CCUG31611 (type strain, equivalent to NCTC12261), whereas *S. pneumoniae* strains included TIGR4, D39, and BHN418 (6B). The BHN418 6B strain was used in Experimental Human Pneumococcal Carriage (EHPC) studies (8). All strains were stored at −80°C in trypticase soy broth (Becton Dickinson, Franklin Lakes, NJ, USA), and 15% glycerol. Before use, stock cultures were diluted 1:10, followed by growth at 37°C to an optical density (OD) of 0.5 at 600 nm in a 5% CO_2_ incubator. The bacterial cells were harvested by centrifugation at 5,000 *g* for 10 min at 4°C and washed in endotoxin free Dulbecco’s-PBS (Sigma-Aldrich, St. Louis, MO, USA) for further processing. To lyse the bacterial cells, a Precellys Lysing Kit and Homogenizer (Precellys 24, Bertin Instruments) was used as per the manufacturer’s instruction. In brief, the bacterial cells dissolved in cold PBS were poured into the Precellys Lysing tube prefilled with high quality glass beads (0.5 mm) and homogenized for disruption of bacterial cells (Precellys 24 homogenizer; program 2). After homogenization, the bacterial cell lysate was centrifuged at 1,000 *g* for 5 min at 4°C and the supernatant was collected and stored at −80°C for further use. The total protein in the cell lysate was quantified using BCA Protein Assay Kit (ThermoFischer Scientific).

### Raising Antisera

*Streptococcus mitis* antisera were obtained from specific pathogen free New Zealand White rabbits (ProteoGenix, France). Rabbits were subcutaneously immunized with 10 × 10^6^ colony-forming units (CFUs) of ultraviolet-killed *S. mitis* type strain (CCUG31611) twice at an interval of 2 weeks, and the antisera were collected at day 28 after the first immunization. We also purchased rabbit antisera raised against whole cell of *S. pneumoniae* serotypes; (1) Type serum 2 against 1, 2, 4, 5, 11, 18, 20, 22, and 33 serotypes (SSI Diagnostica, Denmark) and (2) pooled antisera against *S. pneumoniae* 3, 4, 6, 9, 14, 18, 19, and 23 surface antigens (Meridian Life Sciences, TN, USA), for specific experiments. Pre- and post-inoculation plasma samples from human volunteers intranasally inoculated with *S. pneumoniae* were received from the EHPC collaboration at the Liverpool School of Tropical Medicine, Liverpool, UK (24). In brief, 13 healthy individuals of 18–50 years of age were inoculated with 8 × 10^4^ CFU per 100 µl of *S. pneumoniae* 6B (BHN418) *per naris*, which eventually developed pneumococcal carriage, as previously described (24). Plasma samples were collected at baseline and at day 21 post-inoculation and stored at −80°C (24). The individuals naturally colonized with pneumococcus at baseline or in regular contact with at-risk individuals were excluded. Of note, both informed and written consent was obtained from the participants. Ethical approvals were obtained from the National Health Service Research Ethics Committee (12/NW/0873).

### Western Blot and Immunoinhibition Assay

SDS-PAGE-separated proteins were transferred from gradient gels (4–20%) to nitrocellulose membranes using a constant voltage of 100 V for 2 h using the BioRad Midi-PROTEAN Western blotting module according to the manufacturer’s instructions (BioRad, CA, USA). The membranes were blocked with blocking solution [Tris-buffered saline (TBS) (pH 7.4) containing 0.1% Tween 20 (Sigma-Aldrich) and 5% BSA] for 1 h at room temperature, washed three times with TBS-Tween, and incubated overnight with antisera at 4°C. Following the incubation, the membranes were washed and finally incubated with AP-conjugated goat anti-rabbit or antihuman IgG secondary antibodies and later developed with the substrate (4-chloro-1-naphthol). Of note, the exposure time was similar for the membranes treated with pre- and post-inoculation serum/plasma samples. For immunoinhibition assays, lysates from *S. mitis* CCUG31611 and *S. pneumoniae* 6B were immobilized on CNBr-activated Sepharose 4B beads according to the instructions of the manufacturer (GE Healthcare, Germany). After blocking with 0.2 M glycine (pH 8), the beads were washed and used for absorption of rabbit anti-*S. mitis* antibodies. Following absorption, the unabsorbed serum antibodies were used to perform immunoblotting.

### ELISA

Bacterial whole-cell ELISAs were performed to measure the levels of antibodies. Briefly, each well of a 96-well plate (Maxisorb, Nunc, Thermo Scientific) was coated overnight with 100 µl of bacterial suspension (OD_600_ 0.5), which was washed and then fixed with 10% formalin. The plate was washed four times (200 µl PBS + 0.05% Tween) before addition of 100 µl blocking buffer (PBS + 0.05% Tween + 1% BSA) and incubation for 1 h at 37°C. Serially diluted plasma samples were added to wells in duplicate, incubated for 2 h at room temperature before addition of the anti-IgG/HRP secondary antibody diluted 1:10,000, followed by incubation for 2 h at room temperature, washing and addition of 100 µl of TMB substrate (ThermoFisher Scientific, Rockford, IL, USA) to each well. The plates were then incubated in the dark at room temperature for 15 min, after which stop solution (ThermoFisher Scientific, Rockford, IL, USA) was added to each well to terminate the reaction, and absorbance was measured by reading the plates at 450 nm using a cell imaging Multi-Mode reader (BioTek™ Cytation™ 3; ThermoFischer Scientific). For evaluation, titration curves were established for each measurement and cutoffs applied to convert serum activities into titers. For estimation of titers, cutoffs were placed at OD_450_ = 0.4 for the rabbit hyperimmune sera and OD_450_ = 0.2 for the human sera because those values were within the linear parts of the titration curves. As an example, an ELISA titration curve has been provided as Figure S2 in Supplementary Material.

### Production and Overexpression of Recombinant Proteins

*E. coli* clones for expression of the *S. pneumoniae* TIGR4 recom-binant proteins were generated at John Craig Venter Institute, USA, whereas expression clones for the *S. mitis* type strain recombinant proteins were constructed at the Department of Oral Biology, University of Oslo, Norway. Gateway cloning strategy was used to generate expression clones for the production of recombinant proteins. Briefly, *S. mitis* target genes were amplified using the oligonucleotide primers listed in Table S1 in Supplementary Material. *S. pneumoniae* and *S. mitis* genes cloned in entry clone pDONR221 were transferred into the destination vector T02, which encodes a hexa-histidine tag at the amino terminal as described previously (25). The cloned inserts were sequence validated before transformation of T02 destination vector into *E. coli* expression strain BLR (DE3). For overexpression of recombinant proteins, frozen stock of *E. coli* BLR (DE3)-containing expression clones were inoculated in 1 ml 2× YT medium in deep 96-well blocks with ampicillin (100 µg/ml) and tetracycline (12.5 µg/ml). The bacterial cultures were incubated at 37°C with shaking at 800 rpm. Upon reaching an OD_600_ of 0.7–0.9, protein expression was induced by addition of isopropyl-β-d-1-thiogalactopyranoside (IPTG) at a final concentration of 1 mM, and incubation proceeded overnight at 25°C at 900 rpm. The overnight cultures were then collected by centrifugation at 3,500 rpm at 4°C for 20 min. The pellets were resuspended in 90 µl low salt lysis buffer (50 mM Tris, 100 mM NaCl, 10 mM imidazole, pH 7.3), 1 mM DTT, 1 µl lysonase (Novagen), and 200 µM aminoethyl benzenesulfonyl fluoride hydrochloride (AEBSF) protease inhibitor and incubated for 20 min at room temperature. To complete the lysis process, 10 µl of pop culture (Novagen) was added and incubated for 30 min at room temperature. The bacterial lysate was run on the Nu-PAGE gel (Invitrogen Nu-PAGE 12% Bis–Tris gel) to validate expression and size of proteins.

### On-Chip Purification of Protein Microarray and Testing With Antiserum

High-density Cu^++^ chelated slides (MicroSurfaces, Inc., Englewood, NJ, USA) were used for on-chip purification of hexa-His tagged recombinant proteins as previously described (26). Slides were incubated at room temperature in a humidity chamber for 30 min before printing. Cell lysates were diluted 1:20 in printing buffer PBST (10 mM phosphate buffer, 2.7 mM KCl, 140 mM NaCl, 0.05% Tween 20, pH 7.4) and printed manually on chips with Microcaster array tool (Whatman). Slides were dried in 70% humidity chamber at room temperature. The printed slides were assembled in the fast frame incubation chamber (Whatman) and blocked with blocking buffer (RayBiotech) for 30 min at room temperature. The antisera were cleaned up with *E. coli* lysate and protease inhibitor cocktail by incubation and spinning down. Blocking buffer was removed, the cleaned and diluted 1:100–1:1,000 antisera in blocking buffer were added on the slides and incubated for 1 h. After incubation, the arrayed slides were washed with 1 ml PBST four times by incubating for 2 min between each wash. Alexa-647 conjugated goat anti-rabbit antibody was also cleaned with *E. coli* cell lysate and applied on the slide and the secondary antibody on the array was incubated for 1 h. The arrays were washed with PBST four times for 2 min each wash. The array assembly was departed, and the array slide was put into a 30 ml washing tube and washed with PBST for 15 min. Then, the array slides were rinsed with water and dried by centrifugation. To detect immobilized recombinant proteins, Alexa-555 conjugated anti-hexa-His tag antibody (ThermoFisher Scientific) was cleaned with *E. coli* lysate, diluted to 1:1,000 in blocking buffer, applied on the array slide and incubated for 1 h. After incubating, the array slides were washed and dried as described earlier. Slides were scanned using Genepix 4000B and quantified using Genepix Pro 4.0.

### Purification of PsaA Protein of *S. mitis* and *S. pneumoniae*

The PsaA protein of *S. mitis* and *S. pneumoniae* was purified using Dynabeads TALON which utilizes cobalt based immobilized metal affinity chromatography. *E. coli* BLR (DE3) expression clones containing PsaA gene from *S. mitis* and *S. pneumoniae* were grown in 50 ml 2× YT medium supplemented with ampicillin (100 µg/ml) and tetracycline (12.5 µg/ml). Protein expression was induced by IPTG at OD_600_ 0.6 followed by incubation at 25°C for 4 h at 200 rpm. Cells were harvested by centrifugation, and cell pellets were stored overnight at −80°C. For cell lysis, pellets were resuspended in B-PER complete bacterial protein extraction reagent (ThermoFisher Scientific). Supernatant was collected by centrifugation of cell lysates at 16,000 *g* for 20 min and subjected to His-tag protein purification using Dynabeads TALON (Dynal biotech) according to the manufacturer’s instructions. The purified proteins were analyzed by SDS-PAGE gel electrophoresis, and reactivity to *S. mitis* and *S. pneumoniae* rabbit antisera was assessed using Western blot.

### Statistical Analysis

Wilcoxon Signed Rank tests were applied using the SigmaPlot v13, Systat Software Inc., London, UK. *p* Values < 0.05 were considered to be significant.

## Results

### IgG Antibodies Specific to *S. mitis* Cross-React With Different *S. pneumoniae* Serotypes

**Figure 1:**
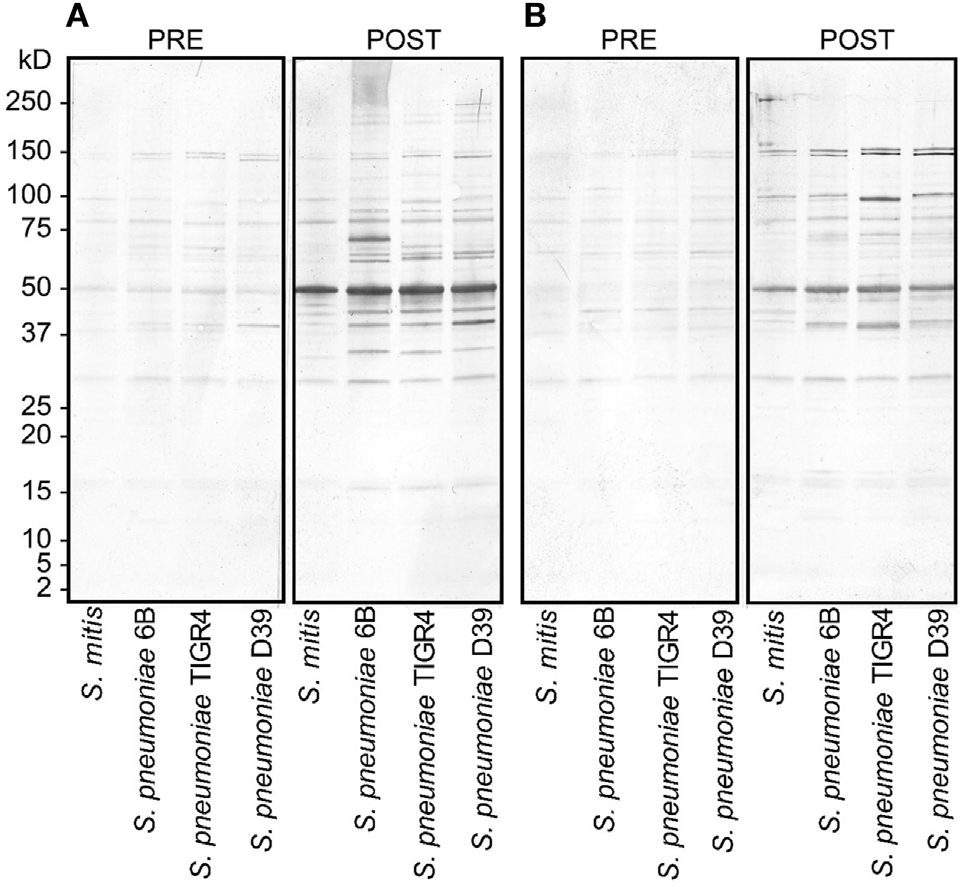
Reactivity of rabbit antisera raised against *Streptococcus mitis* with *Streptococcus pneumoniae* strains (6B, TIGR4, and D39) by SDS-PAGE in gradient gels and immunoblotting. Each lane was loaded with 50 µg of protein from the indicated bacterial species. The pre- and post-inoculation sera were diluted 1:1,000. **(A,B)** Antisera from rabbit-1 and rabbit-2, which were immunized with *S. mitis* CCUG31611. The experiment was performed twice. Abbreviations: PRE, pre-inoculation sera; POST, post-inoculation sera.

**Figure 2:**
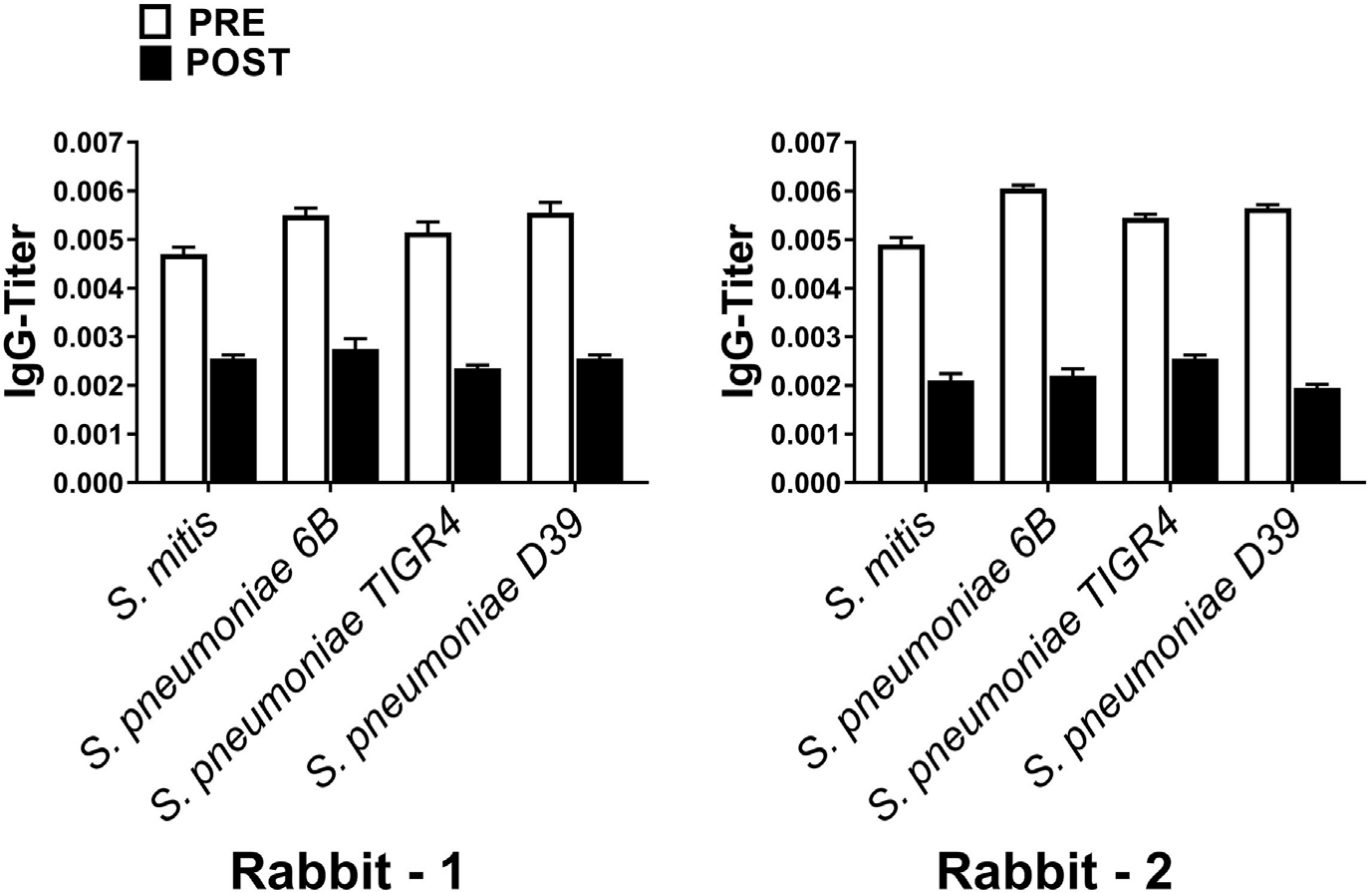
Levels of *Streptococcus pneumoniae*-reactive IgG antibodies from pre- or post *Streptococcus mitis*-immunized rabbits. The titer of IgG antibodies was determined using whole-cell ELISA. Antisera were used from rabbit-1 and rabbit-2, immunized with *S. mitis* CCUG31611. The serum samples were serially diluted, and the OD values converted to titers, with a cutoff of OD value of 0.40 (see Materials and Methods; low numbers indicate high antibody concentration, e.g., 0.002 = 1/500 and 0.004 = 1/250). The bars show averages of two replicates. Abbreviations: PRE, pre-inoculation sera; POST, post-inoculation sera.

**Figure 3:**
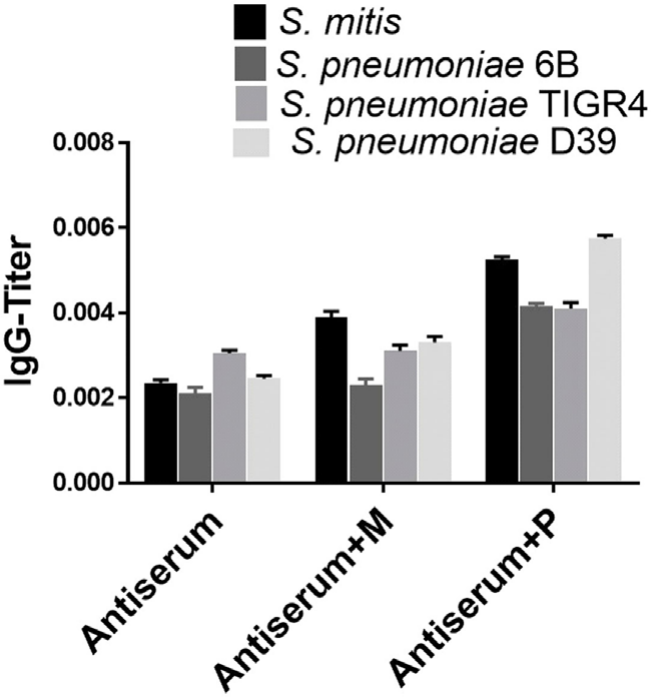
Immunoinhibition for IgG cross-reactivity between *Streptococcus mitis* and *Streptococcus pneumoniae*. For SDS-PAGE, wells were loaded with 50 µg of protein from *S. mitis* CCUG31611 and *S. pneumoniae* strains. After blotting, untreated rabbit *S. mitis* antiserum or the same antiserum pre-incubated with immobilized *S. mitis* or *S. pneumoniae* 6B lysates were used to detect immunoreactivity. Levels of *S. mitis* or *S. pneumoniae*-reactive IgG antibodies in the same antiserum preparations. The levels of IgG antibodies were determined using whole-cell ELISA. The OD values were converted to titers, with a cutoff of OD value of 0.40 (see legend to Figure 2). The bars show averages of two replicates.

To assess the reactivity of antibodies raised against *S. mitis* with pneumococcal antigens, we used antisera from rabbits that received *S. mitis* type strain (rabbit 1 and rabbit-2), and performed SDS-PAGE and immunoblotting. We found an enhanced binding of the IgG antibodies from *S. mitis* antisera (rabbit-1 and rabbit-2), compared with the pre-immunized sera, to the proteins from *S. pneumoniae* serotypes (6B, TIGR4, and D39), as shown by multiple stronger bands (Figures 1A,B). Most of the strongest bands were present at the level of molecular weight 50 kDa for all the *S. pneumoniae* serotypes (Figures 1A,B). Interestingly, a clear and thick band of molecular weight between 50–75 kDa was found in the lane receiving 6B pneumococcal protein, but absent from all other lanes including the one that had *S. mitis* proteins (Figure 1A). Of note, we did not find any appreciable bands while examining the binding of IgA antibodies to pneumococcal proteins (data not shown). After having immunoblotting data on the cross-reactivity of *S. mitis* antisera, we sought to examine the titer of anti-*S. mitis* rabbit IgG antibodies reactive to pneumococcal proteins. We found an increased titer of the IgG antibodies reactive to *S. pneumoniae* and *S. mitis* compared with the antibodies from unimmunized rabbits (Figure 2; *nota bene* as titers were used, lower numbers indicate higher antibody concentration, please see legend). To further confirm the cross-reactivity between *S. mitis* and *S. pneumoniae*, we performed an immunoinhibition assay by incubating rabbit anti-*S. mitis* sera with immobilized *S. pneumoniae* 6B or *S. mitis* lysates. Upon immunoblotting, the *S. mitis* antisera incubated with *S. pneumoniae* 6B or *S. mitis* exhibited significant inhibition of bands for *S. mitis* and *S. pneumoniae* compared with the antisera without antibody absorption (Figure 3). Similar findings were observed when we looked for the bacteria-specific IgG titer in the antisera with different incubations (Figure 3). Overall, these findings show that IgG antibodies specific to *S. mitis* cross-react with *S. pneumoniae*.

### Enhanced Reactivity of Antisera Raised Against *S. pneumoniae* to *S. mitis*

**Figure 4:**
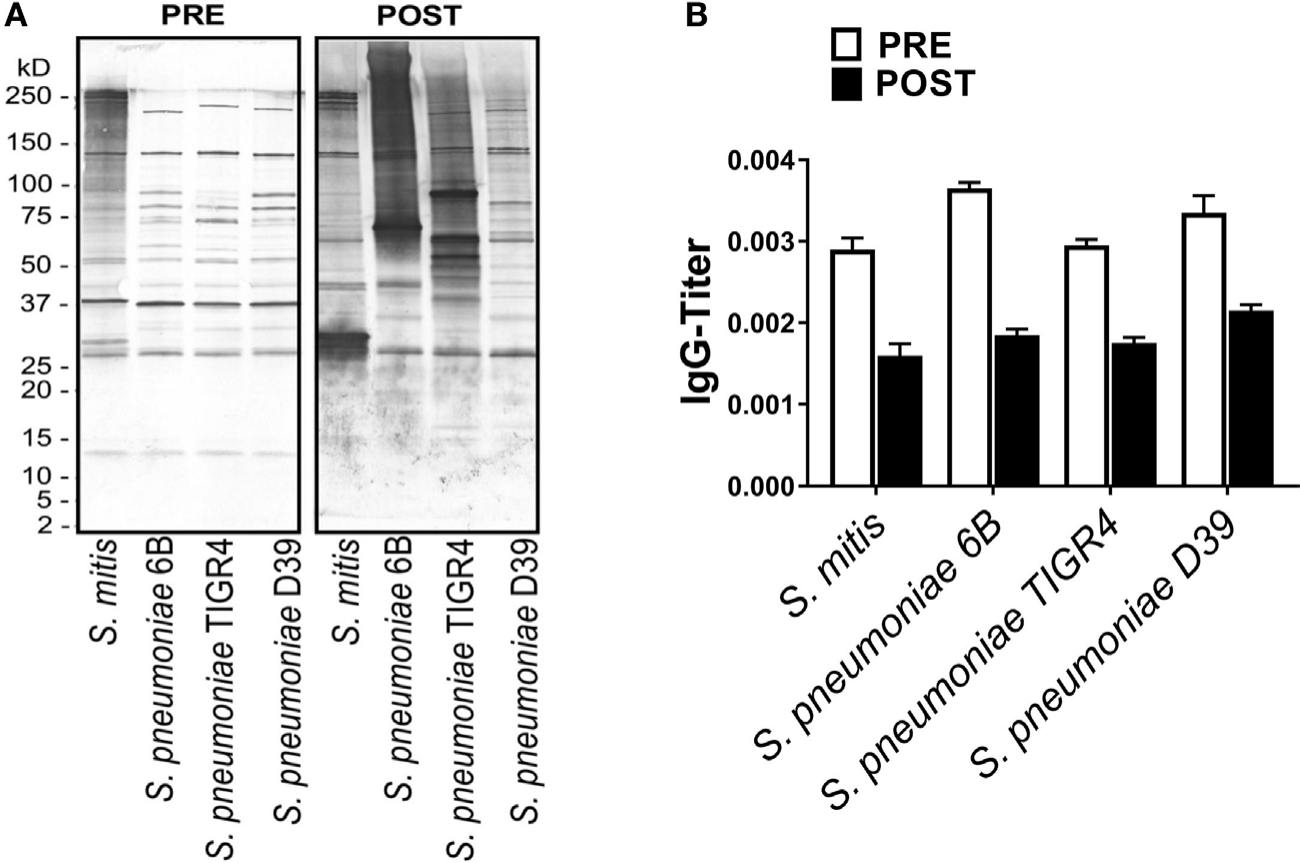
Reactivity of rabbit antiserum raised against *Streptococcus pneumoniae* with *Streptococcus mitis*. **(A)** Reactivity of a rabbit antiserum raised against a pool of *S. pneumoniae* serotypes (1, 2, 4, 5, 11, 18, 20, 22, and 33), with *S. mitis* and *S. pneumoniae* strains (6B, TIGR4, and D39) using SDS-PAGE and immunoblotting. Each lane was loaded with 50 µg of protein from each bacterial species. The pre- and post-inoculation sera were diluted 1:1,000. **(B)** Pre- and post-immunization levels of *S. mitis*-reactive IgG antibodies from a *S. pneumoniae*-immunized rabbit. The titer of IgG antibodies was determined by converting the OD values to titers, with a cutoff of OD value of 0.40 (see legend to Figure 2). The bars show averages of two replicates. Abbreviations: PRE, pre-inoculation sera; POST, post-inoculation sera.

**Figure 5:**
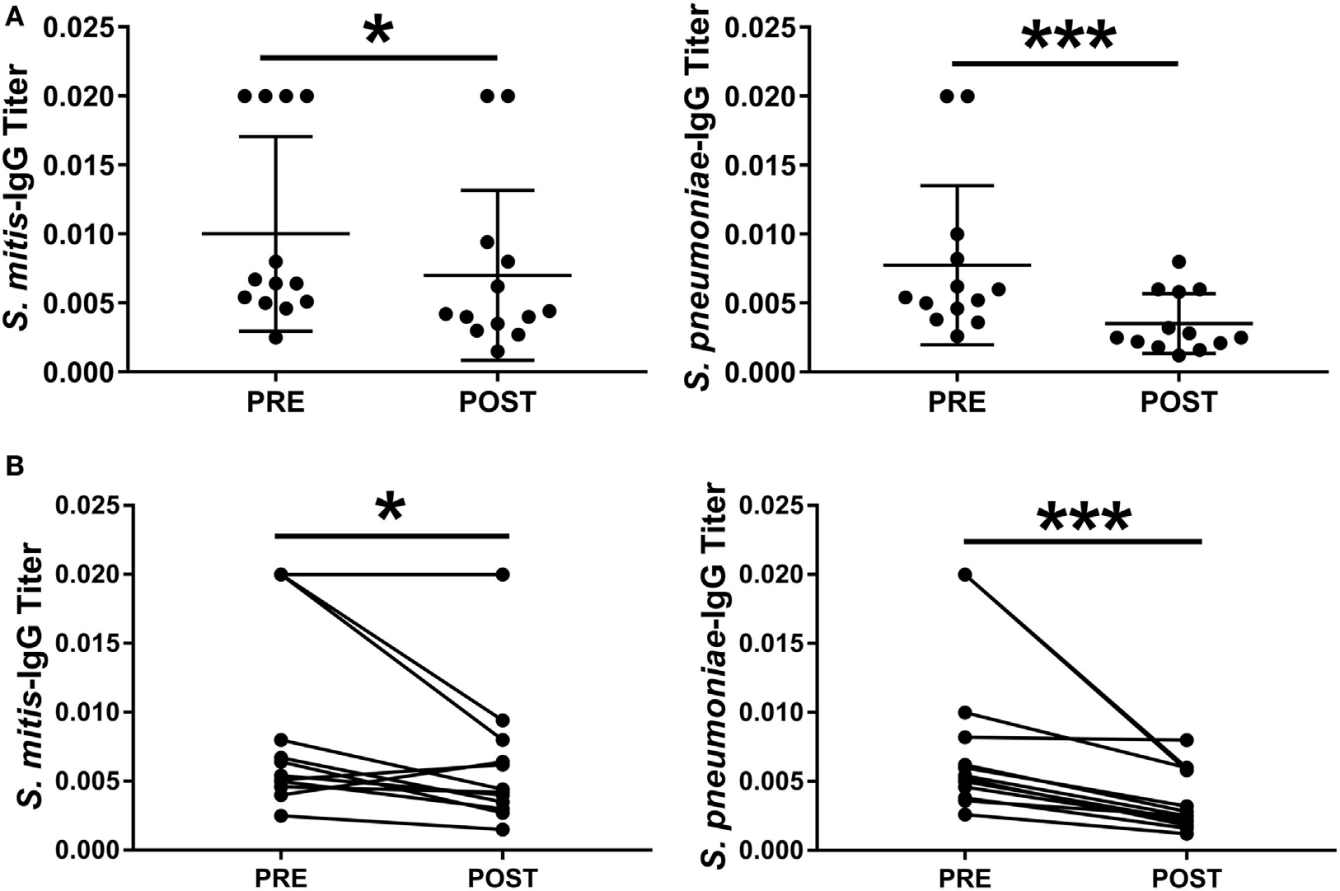
Levels of *Streptococcus mitis*- and *Streptococcus pneumoniae*-reactive IgG antibodies isolated from *S. pneumoniae* 6B-inoculated adults. Pre- and post-inoculation plasma samples were collected from 13 individuals inoculated with *S. pneumoniae* 6B. Serially diluted plasma samples were added to wells in duplicate. The OD values were converted to titers, with a cutoff value of 0.20 (see legend to Figure 2). The levels of IgG antibody titers against *S. mitis* and *S. pneumoniae* strains are shown. OD values are shown for each subject at pre- and post-challenge time points. **(A)** Pre- and post-challenge IgG levels to *S. mitis* and *S. pneumoniae* 6B. **(B)** Pre- and post-challenge IgG levels to *S. mitis* and *S. pneumoniae* 6B linked for each subject. Wilcoxon Signed Rank Test, *p < 0.05; ***p < 0.001. Abbreviations: PRE, pre-inoculation sera; POST, post-inoculation sera.

To understand whether antibodies specific to *S. pneumoniae* can in turn cross-react with *S. mitis*, we used rabbit antisera raised against *S. pneumoniae* to examine the reactivity of the antisera IgG antibodies with *S. mitis* proteins. Our findings demonstrated an enhanced binding pattern of anti-*S. pneumoniae* IgG antibodies to *S. mitis* and *S. pneumoniae* strains, particularly 6B and TIGR4, when compared with the control antibodies from unimmunized rabbits (Figure 4A). However, the post-inoculation binding pattern for *S. mitis* had some differences compared with *S. pneumoniae* 6B and TIGR4 (Figure 4A). Similarly, the levels of anti-*S. pneumoniae* IgG titer reactive to *S. mitis* increased compared with the antibody levels of pre-immunized serum (Figure 4B). To validate these rabbit findings to humans, we used plasma samples from 13 healthy adults intranasally inoculated with *S. pneumoniae* 6B, which eventually acquired pneumococcal carriage (24). Our whole-cell ELISA experiments revealed that plasma IgG antibodies from humans inoculated with *S. pneumoniae* show increased reactivity to *S. mitis* and *S. pneumoniae* 6B compared with the pre-inoculation samples (Figure 5). Taken together, these data indicate that cross-reactivity is conferred by pneumococcal antibodies against *S. mitis* antigens.

### Identification of Pneumococcal Proteins That Cross-React With *S. mitis* Antisera

**Figure 6:**
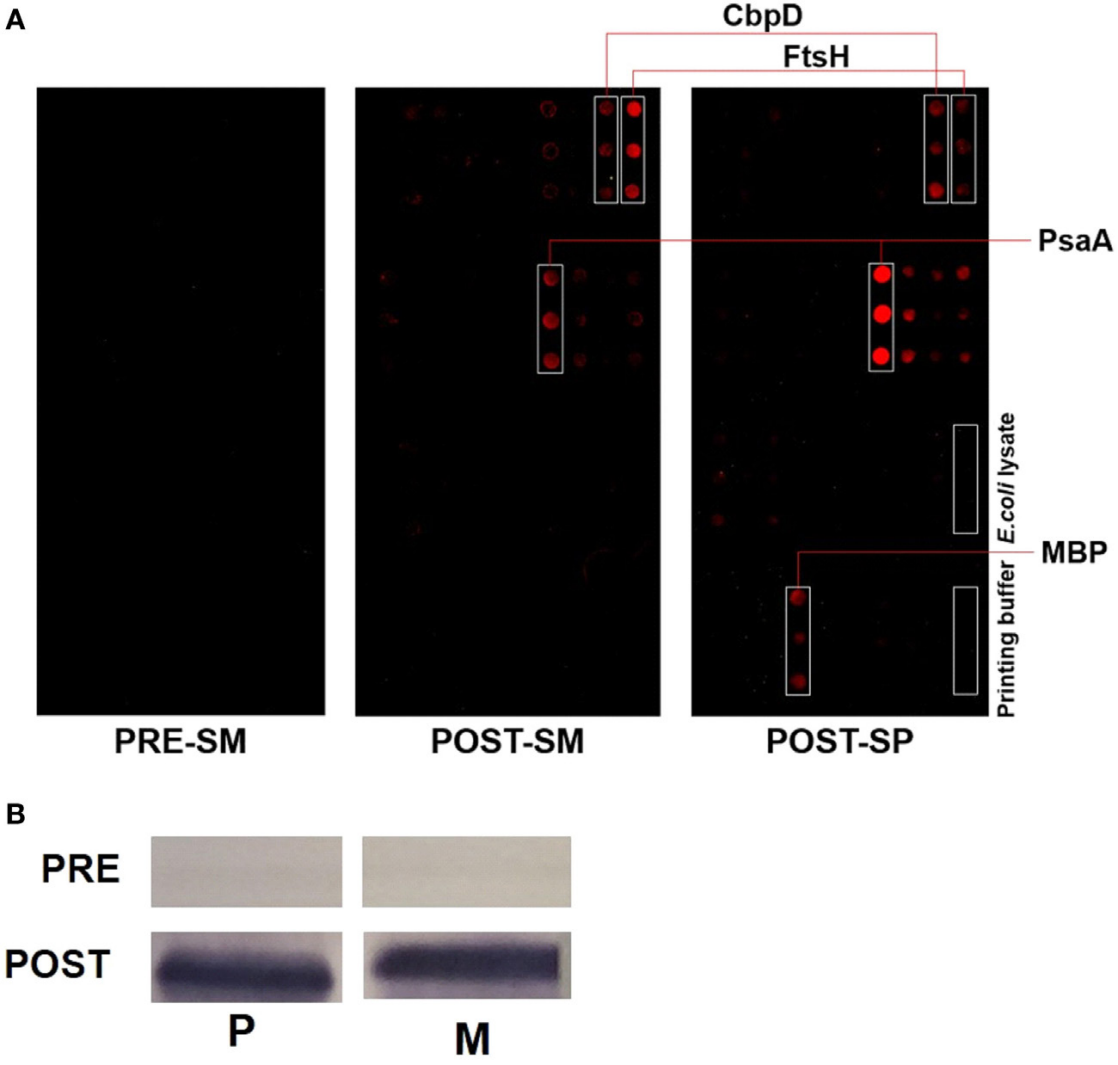
Identification of pneumococcal proteins that cross-react with rabbit *Streptococcus mitis* antisera. **(A)** Immunogenic screening of on-chip purified *Streptococcus pneumoniae* TIGR4 protein microarray. Lysates of 30 recombinant proteins overexpressed in *E. coli* were printed on the chelated Cu^++^/PEG surface slides in triplicates. The slides were probed with *S. mitis* and *S. pneumoniae* antiserum to identify immunoreactive pneumococcal antigens. Pre- and post-immune serum from rabbits challenged with *S. mitis* type strain, and post-immune serum from rabbits challenged with *S. pneumoniae* were used on the slides. Values were obtained by ratio of fluorescence intensity of spot to fluorescence intensity of *E. coli* cell lysate. *E. coli*, His-tag MBP; ~50% similarity to SP2108. Abbreviations: PRE-SM, pre-inoculation *S. mitis* serum; POST-SM, post-inoculation *S. mitis* serum; POST-SP, post-inoculation *S. pneumoniae* serum; CbpD, choline-binding protein D; FtsH, cell division protein; PsaA, manganese ABC transporter adhesion lipoprotein; MBP, maltose-binding protein. **(B)** Reactivity of rabbit anti-*S. mitis* IgG antisera with *S. pneumoniae* TIGR4 and *S. mitis* PsaA by SDS-PAGE in gradient gels and immunoblotting. Each lane was loaded with 50 µg of protein from the indicated bacterial species. The pre- and post-inoculation sera were diluted 1:1,000. Abbreviations: M, *S. mitis* CCUG31611; P, *S. pneumoniae* TIGR4.

Pneumococcal surface proteins have been shown to induce host responses to *S. pneumoniae*, underscoring the potential of these proteins as potent vaccine candidates (6, 7). Our Western blot data show that some of the most prominent bands, which developed due to reaction between *S. pneumoniae*/*S. mitis* proteins and anti-*S. mitis* IgG antibodies had a molecular weight around 50 kDa (Figure 1). To identify some of the bacterial proteins that cross-react with anti-*S. mitis* IgG antibodies, we selected a panel of 30 predicted extracellular and membrane proteins that have around 50 kDa molecular weight (Table 1) and analyzed them using on-chip high quality protein microarray. An array map has been included that shows the position of *S. pneumoniae* TIGR4 proteins printed and immobilized on high-density Cu^++^ chelated slides (Table S2 in Supplementary Material). The immobilized proteins on microarray chips were visualized using Alexa-555 conjugated anti-His-tag antibody. Data analysis identified three different pneumococcal proteins with signal ratio (*F*_sample_/*F_E. coli_*) > 1.5—choline-binding protein D (CbpD; SP2201), cell division protein (FtsH; SP0013), and manganese ABC transporter or manganese-binding adhesion lipoprotein (PsaA; SP1650)—upon treatment with antisera (IgG) against *S. mitis* (Figure 6; Table 2). Maltose/maltodextrin-binding protein (SP2108) was identified using antisera against *S. pneumoniae*, but not with antisera against *S. mitis* (Figure 6A). *S. pneumoniae* PsaA, in particular, is a protein with immunogenic activity that has been investigated in several studies as a potential vaccine against pneumococcal infections (27–29). Thus, we cloned and expressed the *S. mitis* PsaA in *E. coli* to check it for cross-reactivity with antibodies raised against *S. mitis* and *S. pneumoniae*. Our Western blot analysis revealed a stronger binding of rabbit *S. mitis* and *S. pneumoniae* antisera to PsaA compared with the pre-inoculation serum (Figure 6B). The original immunoblot has been provided (Figure S1 in Supplementary Material). Thus, we identified three common proteins, CbpD, FtsH, and PsaA, derived from *S. pneumoniae* reactive to antisera raised against *S. mitis* or *S. pneumoniae*. We also found that purified PsaA from *S. mitis* reacted with the antisera against *S. mitis*, suggesting similar cross-reactive responses for protein antigens from *S. mitis* and *S. pneumoniae*.

**Table 1:** List of *Streptococcus pneumoniae* proteins selected for on-chip protein microarray experiments.

Locus ID S. pneumoniae TIGR4	Protein	Subcellular location	Molecular weight (Da)	Ortholog in Streptococcus mitis NCTC12261	% Identity
SP0013	Cell division protein FtsH	Membrane	71,326	NA^a^	93
SP0091	ABC transporter; permease protein	Membrane	34,545	SM12261_0720	93
SP0092	ABC transporter substrate-binding protein	Extracellular	54,485	SM12261_0719	92
SP0102	Glycosyl transferase	Membrane	40,703	No significant similarity	–
SP0107	LysM domain protein	Extracellular	20,921	SM12261_0703	96
SP0117	Surface protein A	Membrane	82,764	No significant similarity	–
SP0385	Hypothetical protein	Membrane	27,192	SM12261_1022	95
SP0523	ABC transporter, permease protein, putative	Membrane	40,505	SM12261_0538	94
SP0704	Hypothetical protein	Membrane	28,261	No significant similarity	–
SP0749	Branched-chain amino acid ABC transporter; amino acid-binding protein (livJ)	Extracellular	40,414	SM12261_0255	94
SP0751	Branched-chain amino acid ABC transporter; permease protein (livM)	Membrane	33,564	SM12261_0253	96
SP0954	Competence protein CelA	Membrane	23,181	SM12261_1388	92
SP1002	Adhesion lipoprotein (lmb)	Extracellular	33,951	SM12261_0792	93
SP1067	Cell division protein FtsW; putative	Membrane	45,449	No significant similarity	–
SP1069	Conserved hypothetical protein	Extracellular	36,428	SM12261_1418	90
SP1264	Conserved domain protein	Membrane	38,825	No significant similarity	–
SP1400	Phosphate ABC transporter, phosphate-binding protein, putative	Extracellular	31,201	SM12261_0153	78
SP1601	Conserved hypothetical protein	Membrane	24,619	SM12261_0284	92
SP1604	Hypothetical protein	Membrane	16,812	SM12261_0287	79
SP1628	Hypothetical protein	Membrane	8,493	NA	97
SP1650	Manganese ABC transporter; manganese-binding adhesion lipoprotein (PsaA)	Extracellular	34,593	SM12261_0346	95
SP1684	PTS system; IIBC components	Membrane	54,471	SM12261_0375	98
SP1839	ABC transporter ATP-binding protein/permease	Membrane	65,551	NA	92
SP1952	Hypothetical protein	Membrane	36,169	No significant similarity	–
SP1964	DNA-entry nuclease (endA)	Membrane	29,890	SM12261_0490	93
SP2013	Conserved hypothetical protein	Membrane	35,985	No significant similarity	–
SP2051	Competence protein CglC (cglC)	Membrane	12,177	SM12261_0632	91
SP2108	Maltose/maltodextrin ABC transporter maltose/maltodextrin-binding protein	Extracellular	45,337	SM12261_0678	96
SP2201	Choline-binding protein D	Extracellular	50,349	SM12261_0760	89
SP2239	Serine protease	Membrane	41,842	SM12261_0790	94

*^a^NA, not annotated in the NCBI database, but present in the genome sequences*.

**Table 2:** Quantification of fluorescence signal ratio of on-chip purified *Streptococcus pneumoniae* proteins obtained by ratio of fluorescence intensity of spot to fluorescence intensity of *E. coli* lysate.

	*S. pneumoniae*		*Streptococcus mitis*	
	Average	SD	Average	SD
Buffer	0.9	0.1	1.0	0.3
*E. coli*_lysate	1.0	0.2	1.0	0.4
SP0013^a^	2.1	0.4	1.9	0.5
SP0091	1.0	0.1	1.0	0.3
SP0092	1.4	0.2	1.1	0.3
SP0102	1.2	0.2	1.1	0.3
SP0107	0.9	0.1	1.1	0.3
SP0117	2.0	0.4	1.4	0.4
SP0385	1.2	0.2	1.4	0.4
SP0523	1.2	0.2	1.0	0.3
SP0704	1.1	0.3	0.9	0.3
SP0749	1.1	0.1	1.0	0.3
SP0751	1.1	0.1	1.1	0.4
SP0954	1.2	0.2	1.0	0.3
SP1002	1.3	0.2	1.2	0.3
SP1067	1.0	0.2	1.1	0.4
SP1069	1.0	0.1	1.2	0.4
SP1264	2.1	0.4	1.2	0.4
SP1400	1.1	0.1	1.0	0.4
SP1601	1.2	0.2	1.1	0.6
SP1604	1.5	0.2	1.0	0.4
SP1628	1.3	0.2	1.2	0.3
SP1650^a^	41.3	6.4	2.4	0.7
SP1684	1.3	0.2	1.1	0.5
SP1839	1.1	0.1	1.1	0.3
SP1952	1.4	0.3	1.3	0.5
SP1964	1.3	0.4	1.2	0.6
SP2013	1.3	0.1	1.3	0.4
SP2108	2.7	0.9	1.2	0.3
SP2201^a^	2.7	0.6	1.6	0.5
SP2239	1.1	0.2	1.0	0.4

*^a^Signal intensity ratio > 1.5 for both S. pneumoniae and S. mitis*.

*Average values were calculated from three replicates*.

## Discussion

The aim of this study was to examine the antibody-mediated cross-reactivity between *S. pneumoniae* and the commensal *S. mitis* and to identify common cross-reactive protein antigens using multiple experimental approaches. The major findings of the study include the following: (1) rabbit IgG antibodies raised with *S. mitis* cross-react with *S. pneumoniae*; (2) rabbit anti-*S. pneumoniae* IgG antibodies show cross-reactivity with *S. mitis*; (3) IgG antibodies from humans inoculated with *S. pneumoniae* demonstrate increased reactivity to *S. mitis*; and (4) antisera raised against *S. mitis* recognize specific *S. pneumoniae* and *S. mitis* surface proteins.

Our findings demonstrated that antisera raised with *S. mitis* in rabbits cross-react with *S. pneumoniae* serotypes (6B, TIGR4 of different serotypes, and D39). Cross-reactions have been documented among microbial proteins with similar evolutionary development and struc-ture, e.g., antibody cross-reactivity between proteins from the commensal *Neisseria lactamica* and the pathogenic *Neisseria meningitidis* (20, 30). Previous studies suggest that *S. mitis* and *S. pneumoniae* share a close genetic relatedness with around 80% genome similarity, and that several pneumococcal proteins are ubiquitously present in *S. mitis* (31, 32). Therefore, it is understandable why many *S. mitis* and *S. pneumoniae* proteins are reactive to antisera against *S. mitis* as shown in this study. These findings are in line with human studies where salivary antibodies (IgA) were found to react with *S. mitis* (15–17). Since we did not find significant differences between the post-inoculation antibody levels reacting to *S. mitis* and *S. pneumoniae* strains, it may be inferred that the pneumococcal cross-reactive antigens and their ability to bind with *S. mitis* antisera were similar. Another important finding in our study is that IgG antibodies from humans inoculated with *S. pneumoniae* show increased reactivity to *S. mitis* as well as *S. pneumoniae* 6B, as demonstrated by whole-cell ELISA data. This is in accordance with previous studies showing that *S. pneumoniae* carriage promotes an increase in IgG responses specific to protein and capsular antigens of *S. pneumoniae* 6B (8). Overall, these findings support the concept that natural immunity against pneumococcal diseases could be partially due to nasopharyngeal and oral colonization by *S. mitis*, and this may have consequences in the design of better prophylactic strategies for containing infections for all pneumococcal serotypes.

In the on-chip protein microarray analysis, we evaluated 30 different proteins with a molecular weight of around 50 kDa, as major pneumococcal bands in the immunoblotting experiments were found within this range. We identified three pneumococcal proteins that showed reactivity with antibodies (IgG) against both *S. mitis* and *S. pneumoniae*. These proteins included CbpD, PsaA, and FtsH, which are present in the two species and show more than 90% similarity. CbpD and PsaA are extracellular proteins that belong to the family of choline-binding proteins (CBP) and are shown to contribute to nasopharyngeal colonization by *S. pneumoniae* and competence-induced cell lysis (27, 33). So far, several studies on pneumococcal infections conducted in animal models have demonstrated an antibody-mediated protection induced by CBP members, such as PspC and PsaA (27–29). However, the immunogenic potential of CbpD is still unclear. Given the fact that CbpD facilitates nasopharyngeal colonization by *S. pneumoniae* and that it possesses ~90% similarity with its ortholog (SM12261_0760) in *S. mitis*, CbpD holds promise as an immunogenic candidate against *S. pneumoniae* serotypes, warranting further exploration to assess its efficacy to mount protective immunity that is independent of pneumococcal serotypes. A combined formulation of a vaccine containing PsaA and PspA has been shown to prevent bacterial colonization and otitis media in animal models (34, 35). It would be interesting to investigate the immunogenic potential of a combination of PsaA, FtsH, and CbpD, which may confer an additive protective effect as well as limit the occurrence of allelic variation within these individual proteins.

In conclusion, our findings demonstrate that antibodies raised against *S. mitis* and *S. pneumoniae* cross-react with *S. pneumoniae* and *S. mitis* proteins, respectively. These findings are in accordance with our hypothesis that antibodies elicited against *S. mitis* and *S. pneumoniae* show cross-reactivity to *S. pneumoniae* and *S. mitis* proteins, respectively. It is important to note that reactivity of *S. mitis*-specific antibodies with pneumococcal proteins provides a platform upon which the development a serotype-independent protein-based pneumococcal vaccine with extended coverage can be carried out. Future studies need to assess whether antibody responses due to nasopharyngeal colonization by *S. mitis* generate cross-protection against *S. pneumoniae* infections. In addition, it is worth testing the effect of cross-reactive antigens on rendering protection against diverse *S. pneumoniae* serotypes.

## Ethics Statement

Pre- and post-inoculation plasma samples from human volunteers intranasally inoculated with *Streptococcus pneumoniae* were received from the EHPC collaboration at the Liverpool School of Tropical Medicine, Liverpool, UK. Ethical approvals were obtained from the National Health Service Research Ethics Committee, UK (12/NW/0873).

## Author Contributions

SS designed research studies, conducted experiments, acquired and analyzed data, and wrote the paper. RK designed and conducted experiments, acquired and analyzed data, and wrote the paper. DF, EM, and EG provided serum samples from *S. pneumoniae* (6B) inoculated individuals. GR conducted experiments and analyzed data. DF, DB, and KS contributed to the design of experiments and revision of the manuscript. KK designed and conducted experiments, acquired and analyzed data, and revised the manuscript. FP designed research studies, analyzed data, and wrote the paper. All the authors revised and approved the final manuscript.

## Conflict of Interest Statement

The authors declare that the research was conducted in the absence of any commercial or financial relationships that could be construed as a potential conflict of interest.
