# Supplementary figures and images for: Antibodies Reactive to Commensal Streptococcus mitis Show Cross-Reactivity With Virulent Streptococcus pneumoniae Serotypes
Source: Front Immunol. 2018 Apr 16;9:747. doi: 10.3389/fimmu.2018.00747 (PMC5911667; doi:10.3389/fimmu.2018.00747)

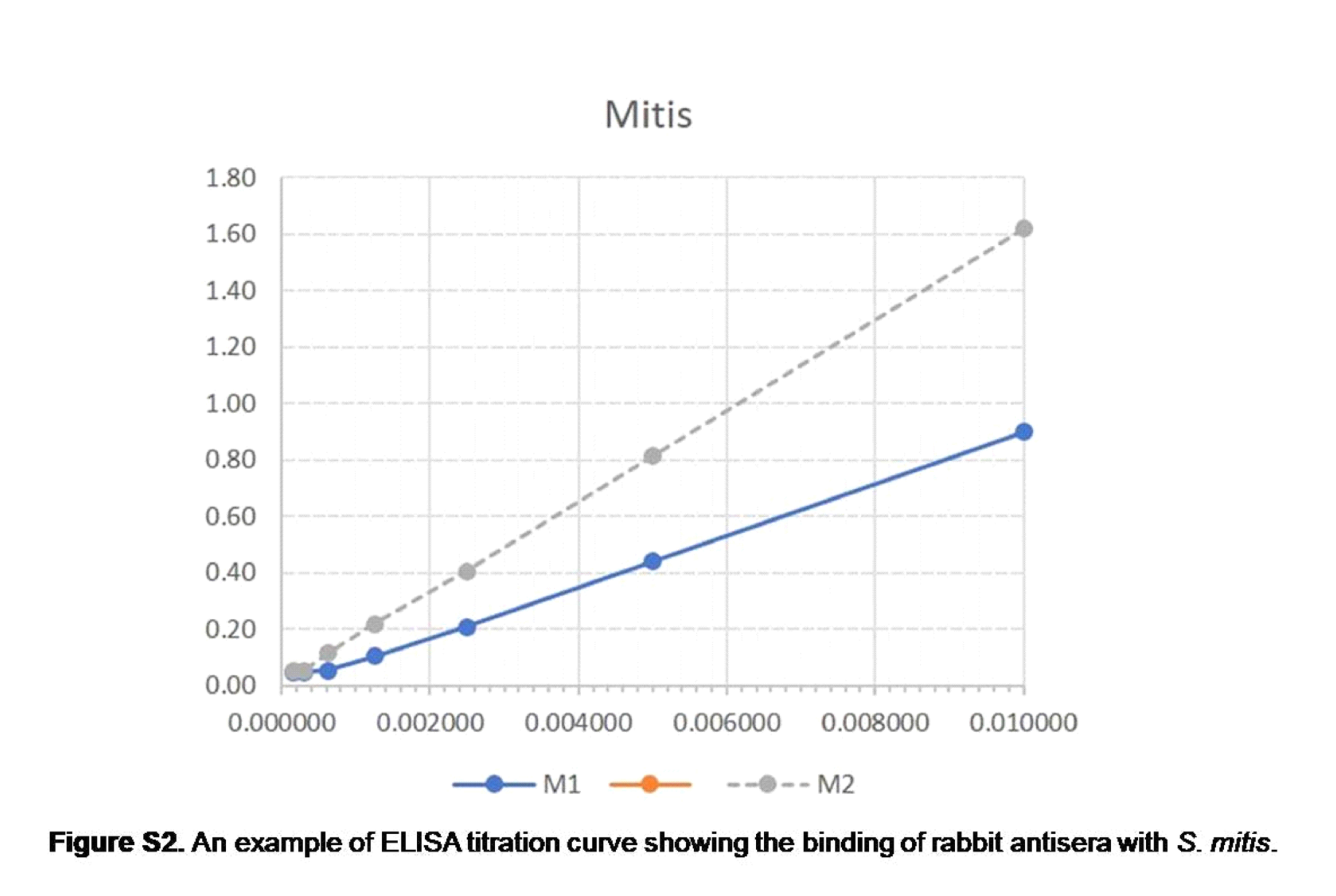

Supplement: Supplementary file 2 [file Image_2.jpg]
