# Supplementary material for: Antibodies Reactive to Commensal Streptococcus mitis Show Cross-Reactivity With Virulent Streptococcus pneumoniae Serotypes
Source: Front Immunol. 2018 Apr 16;9:747. doi: 10.3389/fimmu.2018.00747 (PMC5911667; doi:10.3389/fimmu.2018.00747)
Supplement: Supplementary file 3 [file table_1.docx]

**Supplementary Table 1:** Amplification of S. mitis target genes using the oligonucleotide primers.

| **Nucleotide sequence^a^ (5′ to 3′)** | **Target genes** |
| --- | --- |
| GGGGACAAGTTTGTACAAAAAAGCAGGCTTCATGAAAAATTCACCATTTAAAGTAGC | SM12261_0760  (CbpD) |
| GGGGACCACTTTGTACAAGAAAGCTGGGTCCTATACTCGTTCTCCGTCAC |  |
| GGGGACAAGTTTGTACAAAAAAGCAGGCTTCATGAAAAAATTAGGTACATTATTCGTT | SM12261_0346 (psaA) |
| GGGGACCACTTTGTACAAGAAAGCTGGGTCTTATTTTGCCAATCCTTCAGCAAT |  |
| GGGGACAAGTTTGTACAAAAAAGCAGGCTTCATGAAAAAACAAAATAATGGTTTAATTAAAA | Cell division protein (FtsH)-Not annotated in NCBI database |
| GGGGACCACTTTGTACAAGAAAGCTGGGTCTTATATTTCATCATTCATTTTTGACTTTA |  |

^a^ The attachment site sequence (attB) specific for gateway cloning are underlined.
