# Supplementary material for: Antibodies Reactive to Commensal Streptococcus mitis Show Cross-Reactivity With Virulent Streptococcus pneumoniae Serotypes
Source: Front Immunol. 2018 Apr 16;9:747. doi: 10.3389/fimmu.2018.00747 (PMC5911667; doi:10.3389/fimmu.2018.00747)
Supplement: Supplementary file 4 [file table_2.docx]

| His-MBP^a^ | SP1601 | SP1964 | SP0749 | SP1952 | SP1069 | SP2201 | SP0013 |
| --- | --- | --- | --- | --- | --- | --- | --- |
| His-MBP | SP1601 | SP1964 | SP0749 | SP1952 | SP1069 | SP2201 | SP0013 |
| His-MBP | SP1601 | SP1964 | SP0749 | SP1952 | SP1069 | SP2201 | SP0013 |
| SP1628 | SP0954 | SP1400 | SP2239 | SP1650 | SP1264 | SP0092 | SP0117 |
| SP1628 | SP0954 | SP1400 | SP2239 | SP1650 | SP1264 | SP0092 | SP0117 |
| SP1628 | SP0954 | SP1400 | SP2239 | SP1650 | SP1264 | SP0092 | SP0117 |
| SP1604 | SP0385 | SP1002 | SP1067 | SP0751 | SP0523 | SP1684 | NegCont^b^ |
| SP1604 | SP0385 | SP1002 | SP1067 | SP0751 | SP0523 | SP1684 | NegCont |
| SP1604 | SP0385 | SP1002 | SP1067 | SP0751 | SP0523 | SP1684 | NegCont |
| SP0107 | SP0704 | SP0091 | SP2108 | SP2013 | SP0102 | SP1839 | PB^c^ |
| SP0107 | SP0704 | SP0091 | SP2108 | SP2013 | SP0102 | SP1839 | PB |
| SP0107 | SP0704 | SP0091 | SP2108 | SP2013 | SP0102 | SP1839 | PB |

**Supplementary Table 2:** Array map showing position of *S. pneumoniae* TIGR4 proteins printed and immobilized on high-density Cu^+2^ chelated slides.

Gray shaded boxes show the controls used.

^a^ Positive control for his-tag

^b^ E. coli lysate; Negative control

^c^ PB; Printing buffer
